# The Effect of Season on the Metabolic Profile of the European Clam *Ruditapes decussatus* as Studied by ^1^H-NMR Spectroscopy

**DOI:** 10.3390/metabo7030036

**Published:** 2017-07-26

**Authors:** Violetta Aru, Søren Balling Engelsen, Francesco Savorani, Jacopo Culurgioni, Giorgia Sarais, Giulia Atzori, Serenella Cabiddu, Flaminia Cesare Marincola

**Affiliations:** 1 Chemometrics and Analytical Technology, Department of Food Science, University of Copenhagen, 1165 København, Denmark; se@food.ku.dk; 2 Department of Chemical and Geological Sciences, Cittadella Universitaria di Monserrato, 09042 Monserrato, Italy; 3 Department of Applied Science and Technology (DISAT), Polytechnic University of Turin, 10129 Torino, Italy; francesco.savorani@polito.it; 4 Department of Life and Environmental Sciences, University of Cagliari, 09126 Cagliari, Italy; gsarais@unica.it (G.S.); giul.atzori@gmail.com (G.A.); cabiddus@unica.it (S.C.); 5 Agris-Agricultural Research Agency of Sardinia, Ichthyc Products Research Service S.S. Sassari-Fertilia Km 18,600, 08013 Bonassai, Italy; jculurgioni@agrisricerca.it

**Keywords:** clams, metabolites, NMR spectroscopy, *Ruditapes decussatus*, Santa Gilla lagoon, seasonal change

## Abstract

In this study, the metabolome of *Ruditapes decussatus,* an economically and ecologically important marine bivalve species widely distributed in the Mediterranean region, was characterized by using proton Nuclear Magnetic Resonance (^1^H-NMR) spectroscopy. Significant seasonal variations in the content of carbohydrates and free amino acids were observed. The relative amounts of alanine and glycine were found to exhibit the same seasonal pattern as the temperature and salinity at the harvesting site. Several putative sex-specific biomarkers were also discovered. Substantial differences were found for alanine and glycine, whose relative amounts were higher in males, while acetoacetate, choline and phosphocholine were more abundant in female clams. These findings reveal novel insights into the baseline metabolism of the European clam and represent a step forward towards a comprehensive metabolic characterization of the species. Besides providing a holistic view on the prominent nutritional components, the characterization of the metabolome of this bivalve represents an important prerequisite for elucidating the underlying metabolic pathways behind the environment-organism interactions.

## 1. Introduction

The *omics* techniques (i.e., genomics, transcriptomics, proteomics and metabolomics) are advanced analytical disciplines aimed at the study of pools of biological macromolecules such as DNA (deoxyribonucleic acid), RNA (ribonucleic acid), proteins as well as small metabolites (amino acids, organic acids and sugars) in a given organism. Among them, metabolomics stands out because it provides a reliable snapshot of the physiological downstream status of a biological system. It is focused on the study of the overall low molecular weight metabolites (<1.5 KDa) whose fluxes and levels can vary according to the physiology, development or pathological state of the cell, tissue, organ or organism under investigation [1]. The metabolomics approach has successfully been applied in several fields from nutrition science to ecology where it is used as a valuable monitoring tool of the quality (i.e., chemical and biological contamination) of the environment [2]. In the latter case, the application of metabolomics to the study of the interactions of organisms with their environment, known as environmental metabolomics, has proven to be a powerful analytical tool for assessing organism function and health [3,4,5,6,7,8].

A multitude of biotic and abiotic factors mold the physiology of living organisms. In the case of aquatic organisms, these factors include, but are not limited to, water temperature, salinity, dissolved oxygen and biological factors such as parasites and diseases [9]. An organism’s response to these factors includes multiple behavioral and physiological adaptations that may result in changes in the metabolic features. In this context, the great potential of metabolomics has been shown in several aspects within the seafood processing and production line and some perspectives on the development of innovative metabolomics strategies for the assessment of the health and welfare of wild and cultured aquatic organisms have recently been provided [10,11]. In particular, the metabolomics approach has recently been proposed as a novel analytical tool for biomarker discovery in the aquaculture field since specific molecules can be used to evaluate the pre- and post-harvest quality, food safety and traceability [10,11]. 

Among aquaculture practices, shellfish farming and harvesting have a prominent role in global seafood production and trade. The consumption of marine bivalves (such as oysters, clams and mussels) is further supported by their unique nutritional properties. They are considered an important part of a healthy diet since they are rich in several micro- and macronutrients such as vitamins (vitamins A, B, D and E), minerals (Ca, Mg and Zn) and polyunsaturated fatty acids [12,13]. 

Among bivalve mollusks, the burrowing clam *Ruditapes decussatus* (Linnaeus, 1758) is one of the main shellfish products commercialized in Europe [14]. Several studies, based on traditional analytical methods (such as colorimetric and chromatographic methods), have investigated the metabolic composition of *R. decussatus* in relation to relevant abiotic and biotic factors such as seasonality, food availability and developmental stage [15]. In this work, ^1^H-NMR metabolomics was employed for the first time to assess the seasonal variations in the metabolic profile of wild *R. decussatus.* Clams were collected in the Santa Gilla lagoon (Sardinia, Italy) between May 2013 and July 2014. Water temperature, salinity, dissolved oxygen and pH were measured at the harvesting site. Microscope inspection of the soft tissue, for sex and parasitosis determination, was performed as an integrated part of the metabolomics analysis. The NMR spectral datasets were analyzed using Principal Component Analysis (PCA) [16] and Partial Least Squares Discriminant Analysis (PLS-DA) [17] was employed for the classification of the clams according to sex. 

## 2. Results

The present study was conducted as follows: (i) the water temperature, salinity, dissolved oxygen and pH were measured at the clams harvesting site; (ii) the clams were sampled and sex and parasitosis status were determined by microscopic inspection; (iii) the hydrophilic metabolome of the mollusks was characterized by high resolution ^1^H-NMR spectroscopy; and (iv) multivariate data analysis was employed to explore the metabolic profiles for discriminatory information about season and sex. 

### 2.1. Chemical and Physical Parameters of the Monitored Site

**Figure 1:**
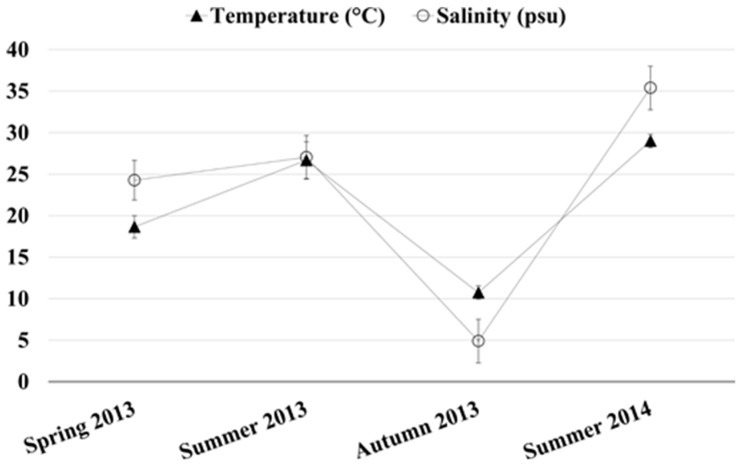
Seasonal values of water temperature (▲) and salinity (o) as measured at the sampling site. Temperature and salinity were measured during each sampling event, namely May 2013 (spring 2013), June 2013 (summer 2013), September and October 2013 (autumn 2013), June and July 2014 (summer 2014). Each measurement was performed in triplicate. Data is reported as mean ± standard deviation.

Dissolved oxygen, pH, temperature and salinity of the water were measured at the sampling site. Among these, the water temperature and salinity values exhibited significant variations over the sampling period, showing the lowest values in October 2013 (10.74 °C, 4.89 psu) and the highest values in July 2014 (28.7 °C, 35.4 psu). The seasonal fluctuations of the above mentioned chemical physical parameters are reported in Figure 1. In contrast, dissolved oxygen did not show any notable seasonal trend and varied from a minimum of 6.19 mg/L measured in July 2014 to a maximum of 9.09 mg/L in October 2013. Similarly, the water pH ranged from 7.4 to 8.9. 

### 2.2. Sex and Parasitosis Determination

**Figure 2:**
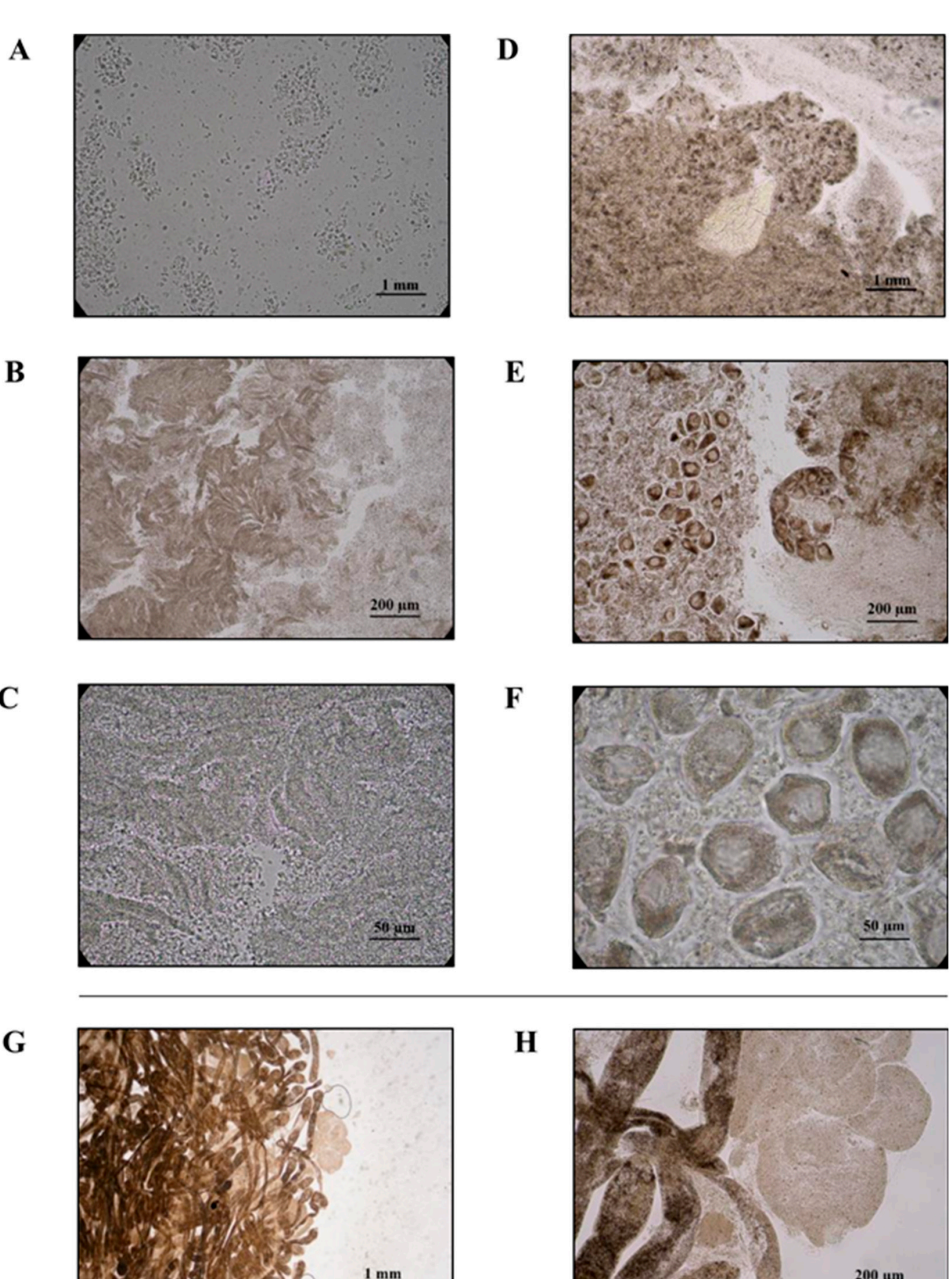
Gonadal tissues of male (left side) and female (right side) *R. decussatus*. Ovary and sperm acini are shown. Magnifications used are 20× (**A**,**D**), 100× (**B**,**E**) and 400× (**C**,**F**) are reported. Gonadal tissues of *R. decussatus* with mother sporocysts of *B. bacciger* containing daughter sporocysts. Magnifications 20× (**G**) and 100× (**H**) are reported. All pictures were taken under a light microscope. Scale bar: 1 mm (**A**,**D**,**G**); 200 µm (**B**,**E**,**H**); 50 µm (**C**,**F**).

Sex was determined through macroscopic inspection of the gonadal tissue, which is composed of a series of compact granular follicles. The follicles were surrounded by connective tissues and contained numerous gametes. Ova and spermatozoa were detected in the clam gonadal tissues enabling all samples to be identified as females or males with an observed sex ratio of 50/50. Figure 2A–F shows representative microscopic views at different magnifications of the ripe female gonads with ova (right-side) and ripe male gonads with spermatozoa (left-side).

The occurrence of parasite infections and tissue degradation was also investigated in the clam samples. Sporocyst and cercariae of the trematode *Bacciger bacciger* (Digenea, Faustulidae) (Rudolphi, 1819) were found in the examined visceral mass (Figure 2G,H) with an infection rate of about 30%. *B. bacciger* is a foodborne helminth pathogen commonly infecting the visceral organs of bivalves [18]. Infection by this parasite has already been reported for several commercial seafoods from the Santa Gilla lagoon including the fish *Atherina boyeri* (Risso, 1810) and the clams *Venerupis aurea* (Gmelin, 1791) and *Ruditapes decussatus* [19]. Since infection by this parasite has been shown to affect the health of clams [20], all samples showing any sign of parasitosis were excluded from any further metabolomic analysis.

### 2.3. NMR Spectra of Clams

**Figure 3:**
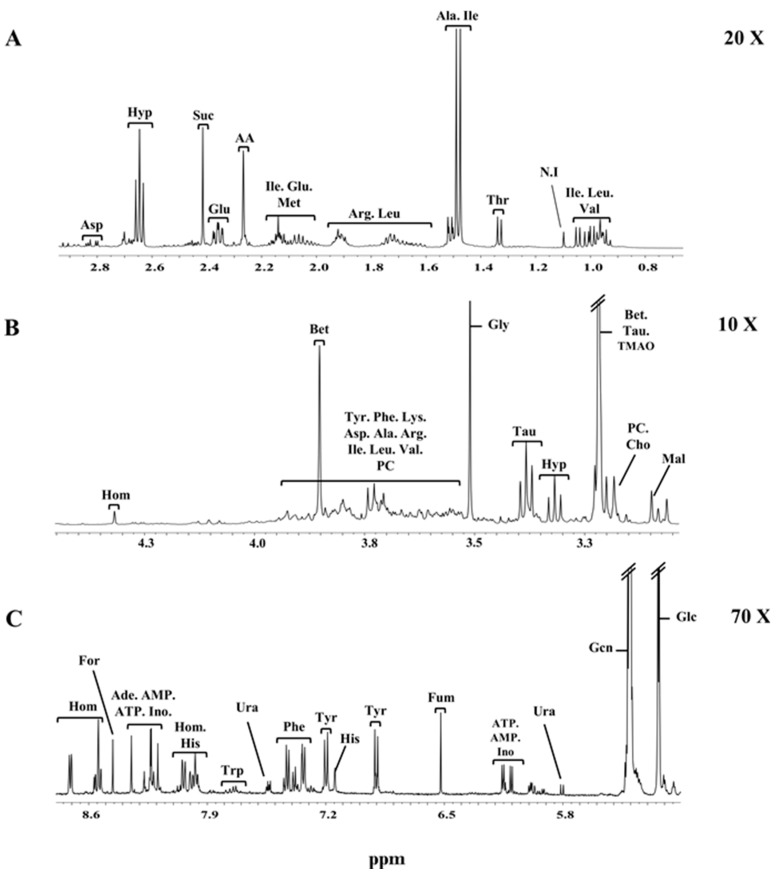
Expansions of the up-field (**A**), mid-field (**B**) and low-field (**C**) regions of a representative proton NMR spectrum of the aqueous extract of clams. Metabolite assignments are given in Table 1.

A representative spectrum of the clams’ hydrosoluble metabolites is shown in Figure 3. The strongest signal in the NMR spectra stems from the ^1^H resonances of trimethylamine N-oxide (TMAO) and betaine (singlet peak at 3.26 ppm). In order to better visualize all signals, as they had very different magnitudes, the NMR spectrum was split into three parts: up-field (Figure 3A), mid-field (Figure 3B) and down-field (Figure 3C) regions. The up-field region between 0.5 ppm and 3.0 ppm is mainly dominated by signals from amino acids and organic acids. In the mid-field region from 3.01 ppm to 5.50 ppm numerous signals arising from carbohydrates and organic osmolytes appear. Among these, the characteristic signals are from the anomeric protons of β-glucose (4.66 ppm) and α-glucose (5.23 ppm), as well as glycogen (5.39 ppm). The organic osmolytes are represented by the typical resonances of betaine (3.25 ppm and 3.89 ppm), hypotaurine (2.64 ppm and 3.34 ppm), taurine (3.25 ppm and 3.44 ppm) and homarine (4.35 ppm). The down-field region (5.50 ppm to 9.00 ppm) was found to be the most heterogeneous spectral region, containing the signals of several organic acids, organic osmolytes, amino acids, nucleotides as well as nucleosides mono- and triphosphate. A total of 34 metabolites were identified using the Biological Magnetic Resonance Data Bank (BMRDB) and by standard addition (spiking). Metabolite assignments, chemical shifts, and multiplicities are reported in Table 1.

### 2.4. Chemometric Analysis

**Figure 4:**
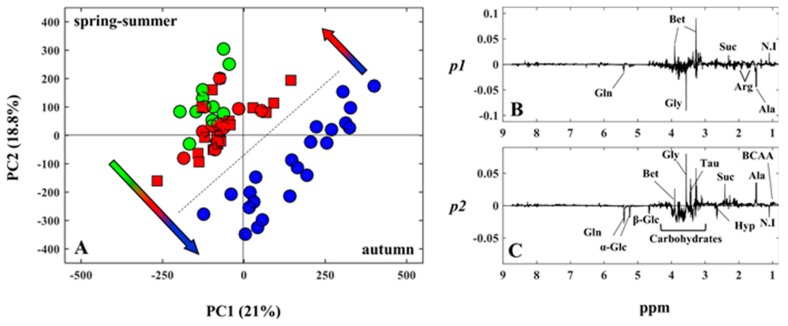
PCA score plot of PC1 versus PC2 (**A**) and PC1 and PC2 loadings plots (**B**,**C**) of the NMR spectral data of clam aqueous extracts. The most significant metabolites are assigned in the loadings plots. Spring 2013 (green circles), summer 2013 (red circles), autumn 2013 (blue circles), summer 2014 (red squares). Arrows are colored according to the chronological course of the experimental seasons.

After visual inspection of the NMR spectra, PCA was performed on the whole spectral dataset in order to investigate the data for sample groupings and trends. Figure 4A shows the scores plot of PC1 versus PC2 (in total, 39% of the systematic variance is explained).

**Figure 5:**
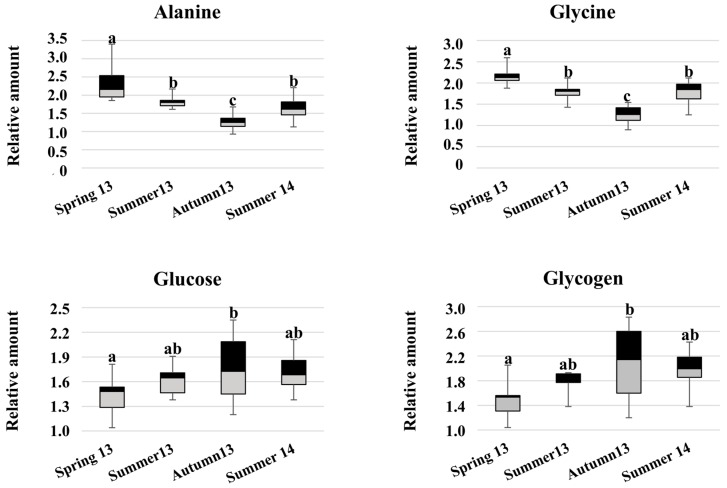
Seasonal fluctuations in the relative amounts of the discriminant metabolites identified in wild samples of *R. decussatus* harvested in the Santa Gilla lagoon. Data is reported as mean ± standard deviation. Different letters stand for significant (*p* < 0.05) differences in the metabolite relative amounts.

A remarkable clustering of the samples into “warm” and “fresh” is readily observed according to the harvesting season. The evolution of the metabolic profiles, according to the chronological course of the experimental seasons, can be shown by following the samples’ distribution from spring (green circles) to summer (red circles) and autumn 2013 (blue circles), back to summer 2014 (red squares). Inspection of the loadings line-plots (Figure 4B,C) enabled the identification of the variables/metabolites that primarily contribute to the observed seasonal pattern. They include free amino acids, organic osmolytes, carbohydrates as well as succinate, an intermediate of the Krebs cycle. The metabolites that mostly influenced sample distribution along the major variation direction PC1 were alanine, glycine, betaine and the unassigned metabolite at 1.08 ppm (Figure 4B), while succinate, carbohydrates (glucose and glycogen) and the amino acids alanine, glycine and taurine were responsible for the scores spreading along PC2 (Figure 4C). The baseline resolved signals of the above-mentioned metabolites were subsequently integrated from the NMR spectra and the respective relative amounts were calculated. Among these, the levels of alanine and glycine decreased simultaneously in autumn 2013 and concomitantly when the highest levels of carbohydrates were found (Figure 5). One-way ANOVA was performed to assess the statistical significance of the inter-group differences. 

**Figure 6:**
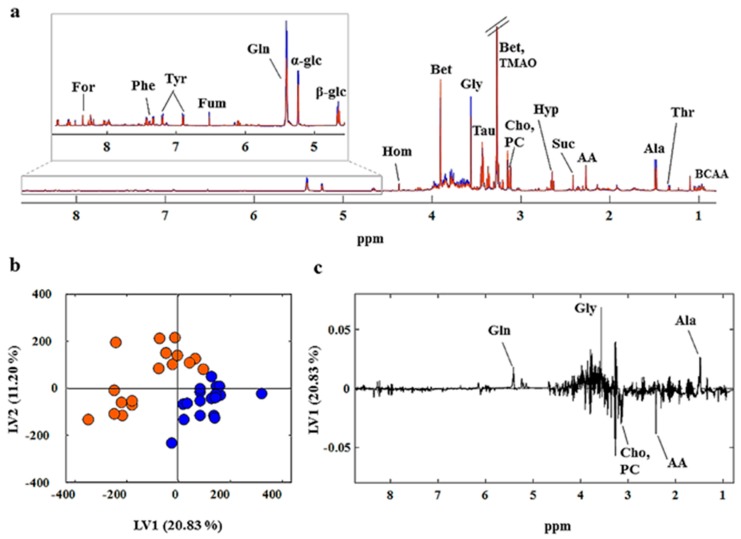
Average spectra of the hydrosoluble extract of male (blue spectra) and female (red spectra) *R. decussatus* (**a**). PLS-DA scores (**b**) and loadings (**c**) plots of the proton NMR spectra of fully ripe clams’ aqueous extracts. Metabolite assignments are reported in Table 1.

The NMR dataset was further scrutinized for possible sex-related differences. Figure 6a shows the average spectra of the hydrosoluble extracts of males (blue line) and females (red lines). A total of 21 metabolites that contributed to the biochemical dissimilarity between males and females were identified in the NMR data by spectral comparison. In male individuals, in particular, the levels of the amino acids threonine, alanine, glycine, and tyrosine, and the carbohydrates glucose and glycogen were found to be higher than in females. In contrast, the metabolic profile of female clams was characterized by higher levels of the organic acids acetoacetate, succinate and formate, the organic osmolytes betaine and taurine, and phosphocholine. However, PCA performed on the dataset did not reveal any strong evidence of sample grouping according to sex. PLS-DA was applied in order to find sex-discriminant metabolites in fully ripe clams. Five metabolites were identified by the PLS-DA model as robust discriminants for sex (Figure 6c). In particular, consistent metabolic differences in the levels of alanine, glycine, choline, phosphocholine and acetoacetate were found between ripe males and females. 

## 3. Discussion

This study investigated the seasonal and sex-related changes in the metabolome of *R. decussatus*, sampled over a period of one year in the Santa Gilla lagoon (Sardinia, Italy), by using a ^1^H-NMR metabolomics approach. PCA, applied to the NMR spectral dataset of the hydrosoluble extract of the clams allowed the identification of several metabolites involved in the harvesting season discrimination. Among these, the amino acids alanine and glycine exhibited significant fluctuations over the sampling seasons and were found to co-vary with the seasonal fluctuations in salinity and temperature, measured at the harvesting site. This result could be explained by the involvement of amino acids in the process of osmotic regulation in bivalves [21]. Indeed, as any other organisms inhabiting transitional water environments (such as lagoons) clams are continuously exposed to salinity oscillations. In particular, the cyclic seasonal temperature variations and the tidal ebb and flow, combined with new freshwater inputs, are among the leading causes behind the salinity changes in shallow-water environments. As any other bivalve species, *R. decussatus* individuals are osmoconformers with little, if any, capability of osmotic regulation of their extracellular fluid (hemolymph) [22]. In these organisms, osmoregulation upon salinity changes relies on the adjustment of the concentrations of intracellular compatible solutes such as free amino acids [23] whose main supply comes from protein-breakdown processes. Even though the physiological rationale behind the observed metabolic regulation is still uncertain, the free amino acids, alanine, arginine, aspartic and glutamic acids, glycine, taurine and betaine, have demonstrated to be particularly important in the process of osmotic regulation in bivalves [22]. 

A second remarkable variation in the clams’ metabolome was observed in the relative increase of the carbohydrate content. Carbohydrates are considered to be the main energy source in marine bivalves since they cover a prominent role in gamete formation as well as in adult survival during nutritive stress periods, such as the reproductive cycle [24]. Thus, the observed seasonal influence on the level of carbohydrates in clams might be associated to changes in the energy metabolism. As reported in the literature, the reproductive cycle in *R. decussatus* involves two phases: a resting phase (autumn-winter) and the gametogenesis (spring-summer) which includes ripeness and spawning [15]. In this study, the highest concentrations of carbohydrates were found in autumn samples while the minimum concentrations occurred in the spring-summer period which match with the clams resting phase and gametogenesis, respectively. The unassigned metabolite found at 1.08 ppm, which exhibited a similar pattern as carbohydrates, may also play an important role in the reproductive cycle. 

PLS-DA analysis of the NMR spectra of the hydrosoluble extract of male and female clams revealed several putative biomarkers for sex discrimination. In agreement with literature concerning other bivalve species, male clams were characterized by significantly higher contents of alanine and glycine [25]. Glycine concentration has been proposed as a biochemical indicator of sex (and maturation) in the sea anemone *Bunodosoma cavernata* (Bosc, 1802) where most of the glycine pool was found in the gonadal tissue of the ripe males [26]. In contrast, choline, phosphocholine and acetoacetate were identified, by the PLS-DA model, as discriminant metabolites for ripe females. Interestingly, previous studies on sex differences in the composition and metabolism of lipids in mussel mantle tissues have demonstrated a lower phosphocholine renewal rate in males compared to female clams [27]. This activity might be correlated with periods of active vitellogenesis in the oocyte of mussels as phospholipids are one of the main storage products of molluscan eggs [27]. 

## 4. Materials and Methods

### 4.1. Chemicals

Analytical grade chloroform (CHCl_3_), methanol (CH_3_OH), deuterium oxide (D_2_O, 99.9%), sodium deuteroxide (NaOD, 40 wt % in D_2_O, 99.5 atom % D), deuterium chloride (DCl, 99 at. % D) and sodium 3-trimethylsilyl-propionate-2,2,3,3-d4 (TSP) were purchased from Sigma-Aldrich (Milan, Italy).

### 4.2. Sample Collection

**Figure 7:**
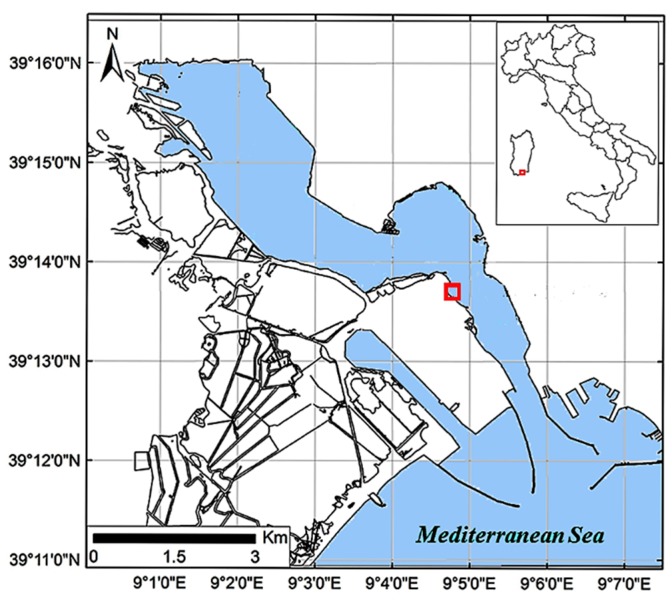
Location of the sampling site in the Santa Gilla lagoon (red square).

The clams were sampled at a commercial harvesting site in the Santa Gilla lagoon (Sardinia, Italy) (Figure 7).

A total of 15 adult specimens (shell length 36.9 mm ± 1.0 mm) were monthly collected by the local fishermen using a shellfish rake (average depth of 1.40 m) in May, June, September and October 2013 and in June and July 2014. After collection, the samples were promptly transported to the laboratory in portable coolers, at approximately 4 °C. In the laboratory, the clams were carefully opened using a stainless steel surgical scalpel blade and the soft tissues were minutely separated from the shell using a spatula. Due to the abundance of lagoonal sediments and soil organic matter in the clams’ gastrointestinal tract, the stomach, digestive gland and intestines were accurately removed by a transverse cut. The remaining tissues were washed with deionized water in order to eliminate any lagoonal residual sediment from the samples. Specimens were dried on blotting paper, put into insulated sterile plastic bags and stored at −80 °C before metabolites extraction.

### 4.3. Water Chemical-Physical Parameters

Water temperature (°C), salinity (psu), dissolved oxygen (mg/L) and pH were measured on each sampling day at the sampling site with a multiparametric probe (Hanna HI 9828).

### 4.4. Sex and Parasitosis Determination

A fresh smear of the gonads, or a fragment of gonadal tissue, was put into a drop of water and compressed between the glass slide and the cover glass. Samples were examined using a Zeiss Primo Star Halogen/LED Microscope. Sex was univocally determined by the presence of ovary follicles in females and gonadal acini in males. Parasitological analysis was performed on the whole tissues using the same methods. 

### 4.5. NMR Analysis

#### Metabolite Extraction

Water-soluble metabolites were extracted according to a modified Folch method [28]. Briefly, each sample was homogenized and a mixture of 12 mL chloroform–methanol (2:1, v/v) was added to 1 g of ground tissues. Four mL of deionized H_2_O was added and the chloroform/methanol/water blend was transferred into a round-bottomed flask and centrifuged at 1700 RCF (Relative Centrifugal Force) for 1 h at 4 °C. The methanol/water mixture was transferred into several 1.5 mL Eppendorf vials and evaporated with an Eppendorf concentrator 5301 (Eppendorf AG, Hamburg, Germany). Samples were subsequently re-dissolved in 1.2 mL of a 0.80 mM D_2_O internal standard solution (TSP) and centrifuged at 13,000 RCF for 5 min at 4 °C to remove particulate matter. The pH of the final sample was adjusted to 6.52 ± 0.03 by adding a small amount of NaOD or DCl to minimize pH-based peak shifts in the NMR spectra. Then, an aliquot of 650 µL was placed into a 5 mm NMR tube for NMR analysis.

### 4.6. NMR Measurements

Proton NMR spectra were acquired with a Varian Unity Inova 500 spectrometer (Agilent Technologies, Santa Clara, CA, USA) operating at 499.84 MHz at 300 K. For each spectrum, 256 scans were collected into 32 k data points over a spectral width of 6000 Hz, with a 45° pulse, an acquisition time of 1.5 s, and a relaxation delay of 4 s. The solvent (water) residual signal was suppressed by applying a presaturation technique with low power radiofrequency irradiation for 1.5 s. An exponential function corresponding to 0.3 Hz was applied to each Free Induction Decay (FID) before Fourier transformation as well as a zero-filling to 65 k data points. NMR spectra were manually phased and the baseline corrected using MestReNova (Version 8.1, Mestrelab Research SL). Spectral chemical shift referencing was also performed in all spectra on the TSP CH_3_ signal at 0.00 ppm. The NMR spectral data were then converted into ASCII, imported into MATLAB R2016a (Mathworks, MA, USA) and a data matrix, sized 60 × 65,536 (samples × variables), was built. 

### 4.7. Preprocessing of the NMR Spectral Data

Prior to multivariate analysis, the whole NMR spectral dataset was corrected for small chemical shift misalignments using the *i*coshift algorithm [29], which performs a correlation optimized shifting of spectral intervals and aligns all the spectra simultaneously. The regions between −0.5 ppm and 0.5 ppm and 4.6 ppm and 5.0 ppm were excluded for multivariate data analysis because of the signals of TSP and water, respectively. After the removal of the noisy region between 9.5 ppm and 10.5 ppm, the final data matrix sized 60 samples × 41,499 variables. NMR spectra were normalized using total area normalization [30]. The dataset was then Pareto scaled, which increases the representation of lower concentration metabolites in the resultant data models while minimizing the contribution from noise [31]. 

### 4.8. Multivariate Analysis of the NMR Spectral Data

Unsupervised exploratory data analysis was performed using Principal Component Analysis (PCA) to show trends, groupings and outliers in the data [16]. PCA is an efficient technique for reduction of data dimensionality and transforms the original variables of the data set into a smaller number of latent variables called Principal Components (PCs), which are uncorrelated with each other and account for decreasing proportions of explained variance. Each principal component is a linear combination of the original variables such that a compact description of the systematic variation within the data set is generated. Most of the information is generally contained in the first few PCs. The results of this exploratory tool can be displayed via scores plots and loadings plots. The loadings show the relation between the original variables and define the direction of the principal components. The scores show the sample variations along the principal components; similar samples will cluster together. 

Partial Least Square Discriminant Analysis (PLS-DA) [17] was applied to investigate biochemical markers for sex discrimination in clams. PLS-DA is a supervised classification tool that is able to find differences among a priori known groups of samples. PLS-DA is usually applied for discriminating between two classes by assigning a “dummy” variable (1 for one class and 0 for the other) to each sample as a reference value in the **Y** dummy matrix in which each column represents a single class. The multivariate data analyses in this study were all performed with the PLS Toolbox 8.1.1 (Eigenvector Research, Manson, WA, USA) and cross validated by means of Venetian blinds sectorization.

## 5. Conclusions

This study has demonstrated the strength of the NMR metabolomics approach for the qualitative and quantitative characterization of the *Ruditapes decussatus* metabolic profile and its changes when exposed to environmental stressors. In particular, the results underline that a main variability in the carbohydrates and free amino acid content is related to seasonal fluctuations in water temperature and salinity. Additionally, the consistent metabolic differences between male and female clams’ metabolic profiles suggest the potential of alanine, glycine, acetoacetate, choline and phosphocholine to act as putative biomarkers for sex differentiation, the first two metabolites being more abundant in male clams, while the latter in ripe female clams.

This study can be considered as a pilot environmental metabolomics study that provides the basis for further investigations aimed at the monitoring of the seasonal fluctuation of the nutritional properties of seafood products. Furthermore, it is demonstrated that the applied non-targeted ^1^H-NMR metabolomics approach can be used as an alternative or complementary approach to the classical and time consuming histological analysis as it includes the biochemical counterpart of sex differentiation.

## Figures and Tables

**Table 1:** List of all the compounds identified in the ^1^H-NMR spectra of the aqueous extract of *R. decussatus*. Chemical shifts and multiplicities used for metabolites assignments are given.

Compound	ppm	Multiplicity *
α-Glucose (α-glc)	5.23	d
β-Glucose (β-glc)	4.66	d
Acetoacetate (AA)	2.27	s
Adenine (Ade)	8.17/8.20	s/s
Alanine (Ala)	1.49	d
Arginine (Arg)	1.63/1.71/1.92	m/m/m
Aspartate (Asp)	2.66/2.80	dd/dd
Adenosine Monophosphate (AMP)	6.13/8.26/8.56	d/s/s
Adenosine Triphosphate (ATP)	6.13/8.25/8.51	d/s/s
Betaine (Bet)	3.26/3.89	s/s
Choline (Cho)	3.19	s
Formate (Form)	8.44	s
Fumarate (Fum)	6.5	s
Glutamate (Glu)	2.36	t
Glycine (Gly)	3.54	s
Glycogen (Gcn)	5.39	bb
Histidine (His)	7.13/7.98	s/s
Homarine (Hom)	4.35	s
Hypotaurine (Hyp)	2.64/3.34	t/t
Inosine (Ino)	6.09/8.22/8.33	d/s/s
Isoleucine (Ile)	0.93/0.99	d/t
Leucine (Leu)	0.96	d
Malonate (Mal)	3.11	s
Methionine (Met)	2.12	s
N.I.	1.08	s
Phenylalanine (Phe)	7.36/7.41/7.42	m/m/m
Phosphoryl Choline (PC)	3.22	s
Succinate (Suc)	2.4	s
Taurine (Tau)	3.25/3.41	t/t
Trimethylamine oxide (TMAO)	3.26	s
Threonine (Thr)	1.32	d
Tryptophan (Trp)	7.72	d
Tyrosine (Tyr)	6.89/7.18	d/d
Uracil (Ura)	5.79/7.52	d/d
Valine (Val)	0.98/1.03	d/d

* s: singlet; d: doublet; t: triplet; dd: doublet of doublets; m: multiplet; bb: broad band; N.I.: not identified.
